# Nonpsychotic Hallucinations and Impaired Vision: The Charles Bonnet Syndrome

**DOI:** 10.7759/cureus.16801

**Published:** 2021-08-01

**Authors:** Francisco J Somoza-Cano, Ahmed Abuyakoub, Faris Hammad, Jasmin Jaber, Abdul Rahman Al Armashi

**Affiliations:** 1 Internal Medicine, St. Vincent Charity Medical Center, Cleveland, USA; 2 Internal Medicine, Northeast Ohio Medical University, Cleveland, USA; 3 Internal Medicine, Hadassah Ein Kerem Hospital, Jerusalem, ISR

**Keywords:** charles bonnet syndrome, release hallucinations, psychosis, glaucoma, reassurance, chronic kidney disease (ckd), heart failure with preserved ejection fraction (hfpef)

## Abstract

Charles Bonnet syndrome is a clinical entity that presents with visual hallucinations in patients with worsening visual acuity and no underlying neuropsychiatric disorder. A 93-year-old male presented to the emergency department complaining of complex visual hallucinations. He had been without his prescription glasses as they were being repaired. After work-up and medication review, no underlying drug or neuropsychiatric disease was found. Moreover, the hallucinations resolved after his vision improved. This case illustrates a frequently missed clinical entity in our practice. Clinical suspicion and reassurance are paramount for adequate patient care.

## Introduction

Charles Bonnet syndrome (CBS) or release hallucinations are visual hallucinations that present in nonpsychotic patients with impaired vision. This syndrome was defined by de Morsier in 1967 [1]. The first report of this condition was of Bonnet's grandparent who had a corneal pathology and started presenting complex visual hallucinations without a psychiatric illness [2]. Current data agree that even though CBS is not exceedingly rare, it is not well recognized by clinicians and may often be misdiagnosed as psychosis or early dementia. The pathophysiology remains highly debatable. However, the term release hallucinations reflects the most concurred pathogenic mechanism. Upon worsening visual acuity, the visual cortical regions become disinhibited, firing spontaneously, producing the hallucinations [3]. They can be as simple as nonformed images, e.g. geometric shapes or light flashes, or complex, where patients report vivid scenes with people and animals on them [4]. Furthermore, the phenomenon is frequently unreported by patients because they fear it may represent an active psychiatric disease, prompting further medical work-up or unwanted social stigma [5]. The standard of care remains correcting the underlying vision disorder, simple behavioral techniques, and reassurance [6].

## Case presentation

A 93-year-old male with a past medical history of bilateral open angle glaucoma, bilateral cataracts status post phakectomy, heart failure with preserved ejection fraction, and chronic kidney disease stage 3A presents to the emergency department after worsening visual hallucinations. The patient reported that four days prior, he started seeing objects move on their own. On admission, he described how he saw the sky turn purple and cars flying through the sky. His insight was intact. Physical examination was grossly within normal limits except for visual acuity of 20/100 on his right eye and 20/50 on his left eye. Montreal Cognitive Assessment Scale (MoCA) was 30. Initial laboratory work, including urine toxicology, was unremarkable besides an elevated creatinine and blood urea nitrogen that were at their baseline (Table 1).

**Table 1:** Laboratory work on admission WBC: white blood cells; RBC: red blood cells; MCV: mean corpuscular volume; MCH: mean corpuscular hemoglobin; MCHC: mean corpuscular hemoglobin concentration; BUN: blood urea nitrogen; AST: aspartate aminotransferase; ALT: alanine transaminase.

Laboratory Finding	Result	Reference Range	Units
Complete Blood Count			
WBC	8.94	3.9 - 11	x10^3^ k/uL
RBC	4	3.5-5.5	x10^6^ k/uL
Hemoglobin	13.5	12-15	g/dL
Hematocrit	41	36-48	%
MCV	93	79-98	fL
MCH	31.3	25.4-34.6	pg
MCHC	35.9	31.5-36.5	g/dL
Platelets	154	140-440	x10^3^ k/uL
Complete Metabolic Panel			
Sodium	141	136-145	mg/dL
Potassium	4.5	3.5-5.1	mg/dL
Chloride	110	98-107	mg/dL
Calcium	8.51	8.5-10.1	mg/dL
Magnesium	1.9	1.6-2.6	mg/dL
Creatinine	1.53	0.55-1.02	mg/dL
BUN	29.9	7-18	mg/dL
AST	17	15-37	U/L
ALT	18	13-61	U/L
Albumin	4	3.4-5.0	g/dL

**Figure 1:**
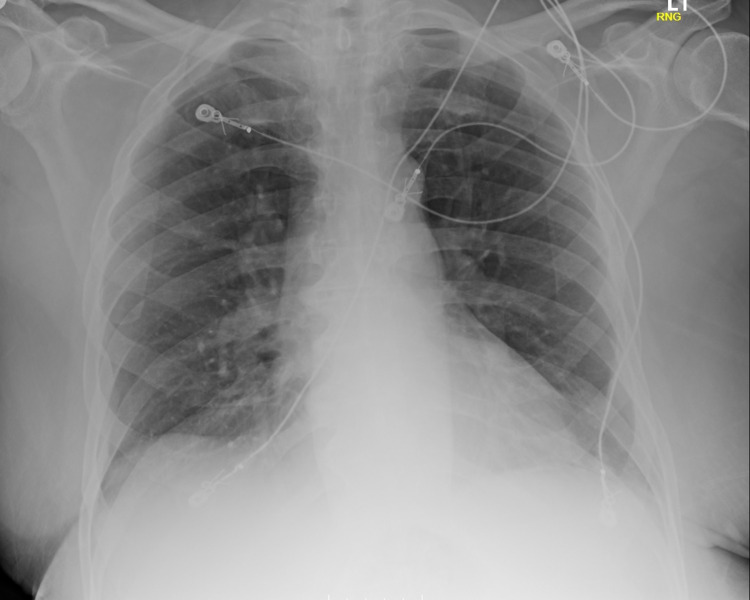
Portable chest X-ray A single anteroposterior chest X-ray was obtained on admission. No acute intrathoracic process was observed. Degenerative change of the spine and shoulders was documented.

The chest X-ray had no acute findings (Figure 1).

A head computed tomography scan had no acute intracranial pathologies. After further work-up and review of medications, no medical causes for acute psychosis were identified. Furthermore, he revealed the hallucinations usually appeared while staring at a white wall and resolved after repetitive blinking. No other sensory modality hallucinations were disclosed. The patient was reassured and instructed to use his prescription glasses continuously. One month later, he presented to the continuity clinic where he denied new hallucinations after his vision improved. 

## Discussion

CBS is not an uncommon occurrence but it is hardly diagnosed by primary care physicians. Intact insight, worsening visual acuity, and visual hallucinations should prompt immediate clinical suspicion. The mechanism behind the hallucinations produced by CBS is yet to be elucidated. The most accepted theory suggests that this condition represents a release phenomenon due to deafferentation of the visual association areas of the cerebral cortex, leading to a form of phantom vision. Moreover, experiments on healthy individuals subjected to visual deprivation report similar occurrences [3,6,7]. 

CBS hallucinations can be variable, ranging from simple figures to clear life-like images, even within the same individual. These hallucinations are usually non-stereotyped, a feature that distinguishes them from epileptic hallucinations. Besides, CBS lacks auditory or other sensory modality hallucinations and does not usually have an emotional impact on the patient. The patients are aware that the phenomenon is not normal as their insight remains unspoiled [6-11].

Furthermore, its defining association is acute or chronic ocular disease. In acute loss of visual acuity, the hallucinations typically present concomitantly as their vision impairment progresses. In chronic ocular disease, a year typically trespasses from the ophthalmological diagnosis and the occurrence of the release hallucinations but the phenomenon can occur at any given time. Most hallucinations resolve between 12 and 18 months [2,4,6,9,10]. 

The most common differential diagnoses include neurodegenerative diseases, migraines with aura, epileptic seizures, and acute psychotic disorders with their underlying causes. Our patient had an MoCA scale of 30 making dementia unlikely [12]. Moreover, he had no history of recent headaches, abnormal movements, or neurological deficits, and his laboratory work-up was grossly unremarkable. Furthermore, CBS differs from these entities by the absence of neurological deficits or psychiatric manifestations and the presence of a known ocular disease as seen in our case. The prognosis is benign and the hallucinations usually resolve within 18 months after the underlying vision deficit improves or is corrected. However, some patients may have persistent release hallucinations especially if they have progressive vision loss. In such cases, the patients should be taught simple behavioral techniques such as rapid eye movement, repetitive blinking, or improving the lighting conditions to repress the hallucinations. Reassurance is of utmost importance to avoid overtreatment [6,13].

## Conclusions

CBS or release hallucinations present in patients with worsening visual loss without a neuropsychiatric illness. Clinical suspicion is paramount for prompt clinical assessment. After the vision disorder is tackled, the hallucinations typically resolve, but persistent hallucinations improve with simple behavioral techniques. Reassurance is critical for preventing excessive medical work-up and avoiding unnecessary patient distress.
